# Genotype by environment interaction using AMMI model and estimation of additive and epistasis gene effects for 1000-kernel weight in spring barley (*Hordeum vulgare* L.)

**DOI:** 10.1007/s13353-019-00490-2

**Published:** 2019-03-15

**Authors:** Jan Bocianowski, Tomasz Warzecha, Kamila Nowosad, Roman Bathelt

**Affiliations:** 1 Department of Mathematical and Statistical Methods, Poznań University of Life Sciences, Wojska Polskiego 28, 60-637 Poznań, Poland; 2 Department of Plant Breeding and Seed Science, University of Agriculture in Krakow, Łobzowska 24, 31-140 Kraków, Poland; 3 Department of Genetics, Plant Breeding and Seed Production, Wrocław University of Environmental and Life Sciences, Grunwaldzki 24A, 53-363 Wrocław, Poland

**Keywords:** *Hordeum vulgare* L., 1000-kernel weight, AMMI, Stability, Genetic parameters, Doubled haploid lines

## Abstract

The objective of this study was to assess genotype by environment interaction for 1000-kernel weight in spring barley lines grown in South Poland by the additive main effects and multiplicative interaction model. The study comprised of 32 spring barley (*Hordeum vulgare* L.) genotypes (two parental genotypes—breeding line 1 N86 and doubled haploid (DH) line RK63/1, and 30 DH lines derived from F_1_ hybrids), evaluated at six locations in a randomized complete block design, with three replicates. 1000-kernel weight ranged from 24.35 g (for R63N/42 in 2011) to 61.46 g (for R63N/18 in 2008), with an average of 44.80 g. AMMI analyses revealed significant genotype and environmental effects as well as GE interaction with respect to 1000-kernel weight. In the analysis of variance, 16.86% of the total 1000-kernel weight variation was explained by environment, 32.18% by differences between genotypes, and 24.50% by GE interaction. The lines R63N/61, R63N/22, and R63N/1 are recommended for further inclusion in the breeding program because their stability and the highest averages of 1000-kernel weight. The total additive effect of all genes controlling the trait and the total epistasis effect of 1000-kernel weight were estimated. Additive gene action effects based on DH lines were always larger that this parameter estimated on the basis of parental lines. Estimates of additive gene action effects based on the all DH lines were significantly larger than zero in each year of study. Epistasis effects based on all DH lines were statistically significant in 2011 and 2013.

## Introduction

Environmental conditions might possess various influence on genotype; therefore, certain genotype responses could differ depending on various environment-forming genotype-by-environment (GE) interaction. The phenotypic presentation of different genotypes could be constant in various environments, whereas some others expose significant variation over diverse environments. The difference between the phenotypic, experiential assessment and the value predictable from the theoretical model of observations that takes into account the general mean as well as genotypic and environmental main effects can be defined statistically as GE interaction. Numerous phenotypic characteristics determined in multi-environment studies, for example yield and its items, expose variation in presentation in different environmental settings; consequently, cultivars may be classified as unstable. The various performance of cultivars might be cleared up with environmental main effect utilization, while the mean values of studied characteristics of all genotypes considerably differ amid environments, and partly can be created by GE interactions, while the differences among genotypes are unequable throughout environments. Breeder and farmers desire constant cultivars or cultivars only slightly modified by environment. Genotype stability or instability might be estimated with a series of trial application. Numerous authors have proposed statistical methods for estimating the manner of genotype reaction to diverse environmental condition (Neyman 1932; Yates and Cochran 1938; Mather and Jones 1958; Finlay and Wilkinson 1963; Eberhart and Russell 1966; Wricke and Weber 1986). The reaction of certain genotypes in diverse environments could be determined in series of trials with application of methods generated by Kaczmarek (1986) and Caliński et al. (1987). Based on above methods, GE effect connected with every genotype (computed by the assessment of the adequate *F*-statistic) is the assessment of stability, and the regression of the GE interaction effects on the tentative means originated from various environments (assessed by the value of the adequate *F*-statistic) is the estimation of adaptability. The calculation of stability articulated in GE interaction with the *F*-statistic value application can be traced back to the methods initially developed by Caliński (1960) and in parallel by Wricke (1962), whereas the *F*-statistic for the regression, measured as the determination of adaptability, is connected to the idea of Finlay and Wilkinson (1963) and Eberhart and Russell (1966). Barley (*Hordeum vulgare* L.) after rice, wheat and maize are fourth cereal crops in the world (https://www.statista.com/statistics/263977/world-grain-production-by-type/ accessed 21.06.2018). Even if it is acknowledged to be acclimated to a wide type of environmental surroundings (e.g., MacGregor and Bhatty (1993)) numerous research have showed a significant impact of environmental conditions and GE interaction on phenotypic presentation of agronomically essential features (Eagles et al. 1995; Kaczmarek et al. 1999; Chełkowski et al. 2000; Warzecha et al. 2011). Barley yield and its structure, as well as other traits connected with barley kernel utilization as food and feed, might be affected by environmental conditions divided into abiotic factors (temperature, precipitation, water contamination, air pollution, etc.) and biotic factors (fungal, viral bacterial pathogen infection, pest damage). For instance, harmful diseases caused by the *Fusarium* species in barley worldwide like *Fusarium* seedling blight (FSB) and *Fusarium* head blight (FHB) (Warzecha et al. 2011; Marin et al. 2013; Nielsen et al. 2014). Grain yield and its quality reduction are caused by the disease as a result of contamination with mycotoxins, which are responsible for mycotoxicoses in humans and domestic animals (Buerstmayr et al. 2009; Marin et al. 2013). The malting and brewing industries also suffer because of contaminated grains (Desjardins 2006; Ma et al. 2009). Response of genotypes on unstable abiotic factors can be assessed by performing research within a number of years and/or in diverse localization, while the result of biotic stresses (e.g., viral, bacterial, or fungal pathogens) could be studied predominantly with artificial infection application.

One of the major aims in spring barley breeding has constantly been rising 1000-kernel weight as a way of increasing yield. An enhanced awareness of genetic determination of 1000-kernel weight can help the breeders to manage the genetic improvement for the crop. 1000-kernel weight is a very complex quantitative feature, and its expression is controlled also in complex way as the result of genotype, environmental factors, and the GE interaction. Complexity of 1000-kernel weight is a consequences of diverse response of genotypes on unstable environmental conditions during plant growth. The GE interaction is frequently analyzed by the additive main effects and multiplicative interaction (AMMI) model (Zobel et al. 1988). The AMMI model combines the analysis of variance for the genotype and environmental main effects and the principal component analysis (PCA) with multiplicative indices in a particular single analysis.

The objectives of this study were (1) to assess genotype by environment interaction for 1000-kernel weight in spring barley (*Hordeum vulgare* L.) grown in South Poland by the AMMI model and (2) to estimate the parameters connected with the additive and additive-by-additive interaction (epistasis) gene action.

## Materials and methods

The material for the studies covered 32 spring barley (*Hordeum vulgare* L.) genotypes: two parental genotypes (breeding line 1N86 and DH line RK63/1 derived from barley cultivars Roland and Kristal), and 30 DH lines derived from F_1_ hybrids. DH lines were developed by *Hordeum bulbosum* technique. Standard procedures were applied for crossing *H. vulgare* with *H. bulbosum* and in vitro culture of immature embryos (Kasha and Kao 1970; Devaux 1986).

Field experiments were carried out over 6 years: 2008–2013 at Prusy, South Poland (near Kraków, 50° 06′ 52″ N, 20° 04′ 23″ E). During each year, the experiment with *g* = 32 genotypes was carried out in a randomized block design, with three replications. In each plot, seeds were sown in six rows 2 m long, 20 cm apart, with each row containing 200 seeds. At full maturity, spikes were harvested manually and 1000-kernel weight was examined. Mean values of temperature and precipitation for seasons of experiment in Prusy near Kraków are presented in Table 1. A two-way fixed effect model was fitted to determine the magnitude of the main effects of variation and their interaction on 1000-kernel weight. Least squares means were simultaneously produced for the AMMI model. The model first fits additive effects for the main effects of genotypes (G) and environments (E) followed by multiplicative effects for GE interaction by principal component analysis. The traditional AMMI model for fixed effects (Gauch and Zobel 1990; Nowosad et al. 2016) is given by:

$$ {y}_{ge}=\mu +{\alpha}_g+{\beta}_e+\sum \limits_{n=1}^N{\lambda}_n{\gamma}_{gn}{\delta}_{en}+{Q}_{ge}, $$

where *y*_*ge*_ is the 1000-kernel weight mean of genotype *g* in environment *e*, *μ* is the grand mean, *α*_*g*_ is the genotypic mean deviations, *β*_*e*_ is the environmental mean deviations, *N* is the number of PCA axis retained in the adjusted model, *λ*_*n*_ is the eigenvalue of the PCA axis *n*, *γ*_*gn*_ is the genotype score for PCA axis *n*, *δ*_*en*_ is the score eigenvector for PCA axis *n*, and *Q*_*ge*_ is the residual, including AMMI noise and pooled experimental error. Expected distribution of *Q*_*ge*_ is normal.

**Table 1:** Meteorological data for seasons of experiments (mean value)

	Temperature (°C)		Precipitation (mm)	
Year	June	July	June	July
2008	18.5	19.1	25.9	142.1
2009	16.1	20.1	163.3	71.67
2010	17.6	20.8	135.1	105.2
2011	18.1	17.7	44.4	194.4
2012	17.7	20.5	143.1	68.7
2013	17.6	19.2	213.1	27.2
Mean	17.6	19.6	120.8	101.5

Source: Date of the Department of Crop Production, Agricultural University in Kraków, Substation Prusy 50.13333° N, 20.0833° E

The AMMI stability value (ASV) was used to compare the stability of genotypes as described by Purchase et al. (2000):

$$ ASV=\sqrt{{\left[\frac{S{S}_{IPCA1}}{S{S}_{IPCA2}}\left( IPC{A}_1\right)\right]}^2+{\left( IPC{A}_2\right)}^2}, $$

where *SS* is the sum of squares, *IPCA*1 and *IPCA*2 are the first and the second interaction principal component axes, respectively; and the *IPCA*_1_ and *IPCA*_2_ scores were the genotypic scores in the AMMI model. ASV is the distance from zero in a two-dimensional scatterplot of *IPCA*_1_ scores against *IPCA*_2_ scores. Since the *IPCA*_1_ score contributes more to GE sum of square, it has to be weighted by the proportional difference between *IPCA*_1_ and *IPCA*_2_ scores to compensate for the relative contribution of *IPCA*_1_ and *IPCA*_2_ total GE sum of squares. The higher the IPCA score, either negative or positive, the more specifically adapted a genotype is to certain environments. Lower ASV score indicates a more stable genotype across environments.

Genotype selection index (GSI) was calculated for each genotype which incorporates both mean 1000-kernel weight and ASV index in single criteria (GSI_i_) as (Farshadfar and Sutka 2003):

$$ {\mathrm{GSI}}_{\mathrm{i}}={\mathrm{RY}}_{\mathrm{i}}+{\mathrm{RASV}}_i, $$

where GSI_i_ is genotype selection index for *i*th genotype, RY_i_ is rank of mean 1000-kernel weight for *i*th genotype, RASV_i_ is rank for the AMMI stability value for the *i*th genotype.

Estimation of the additive gene effect and additive-by-additive interaction of homozygous loci (epistasis) effect on the basis of phenotypic observations requires identification of groups of extreme DH lines, i.e., lines with the minimal and maximal expression of the observed trait (Choo and Reinbergs 1982). The group of minimal lines consists of the lines which contain, theoretically, only alleles reducing the value of the trait. Analogously, the group of maximal lines contains the lines which have only alleles increasing the trait value. In this paper, we identify the groups of extreme lines using the quantile method (Bocianowski et al. 1999), in which lines with the mean values smaller (bigger) than 0.03 (0.97) quantile of the empirical distribution of means are assumed as minimal (maximal) lines. The chosen quantiles 0.03 and 0.97 are the results of previously study (Bocianowski et al. 1999). The total additive effect *a*_*DH*_ of all genes controlling the trait and the total additive-by-additive interaction effect *aa*_*DH*_ may be estimated by the formulas (Bocianowski and Krajewski 2009; Bocianowski 2012b):

$$ {\widehat{a}}_{DH}=\frac{1}{2}\left({\overline{L}}_{\mathrm{max}}-{\overline{L}}_{\mathrm{min}}\right) $$

and

$$ {\overset{\wedge }{aa}}_{DH}=\frac{1}{2}\left({\overline{L}}_{\mathrm{max}}+{\overline{L}}_{\mathrm{min}}\right)-\overline{L}, $$

where ${\overline{L}}_{\mathrm{min}}$ and ${\overline{L}}_{\mathrm{max}}$ denote the means for the groups of minimal and maximal DH lines, respectively, and $\overline{L}$ denotes the mean for all DH lines. Additionally, the additive effects were estimated on the basis of parental observations:

$$ {\widehat{a}}_{Parents}=\frac{1}{2}\left({P}_1-{P}_2\right), $$

where *P*_1_ and *P*_2_ are the means for better- and lower-scoring parents, respectively. The test statistics to verified hypotheses about genetic parameters different than zero are given by:

$$ {F}_a=\frac{M{S}_a}{M{S}_e}\ \mathrm{and}\ {F}_{aa}=\frac{M{S}_{aa}}{M{S}_e}, $$

where *MS*_*a*_ denotes mean square for parameter *a*, *MS*_*aa*_ denotes mean square for epistasis *aa*, *MS*_*e*_ denotes mean square for residual.

All the analyses were conducted using the GenStat v. 18 statistical software package.

## Results

**Fig. 1:**
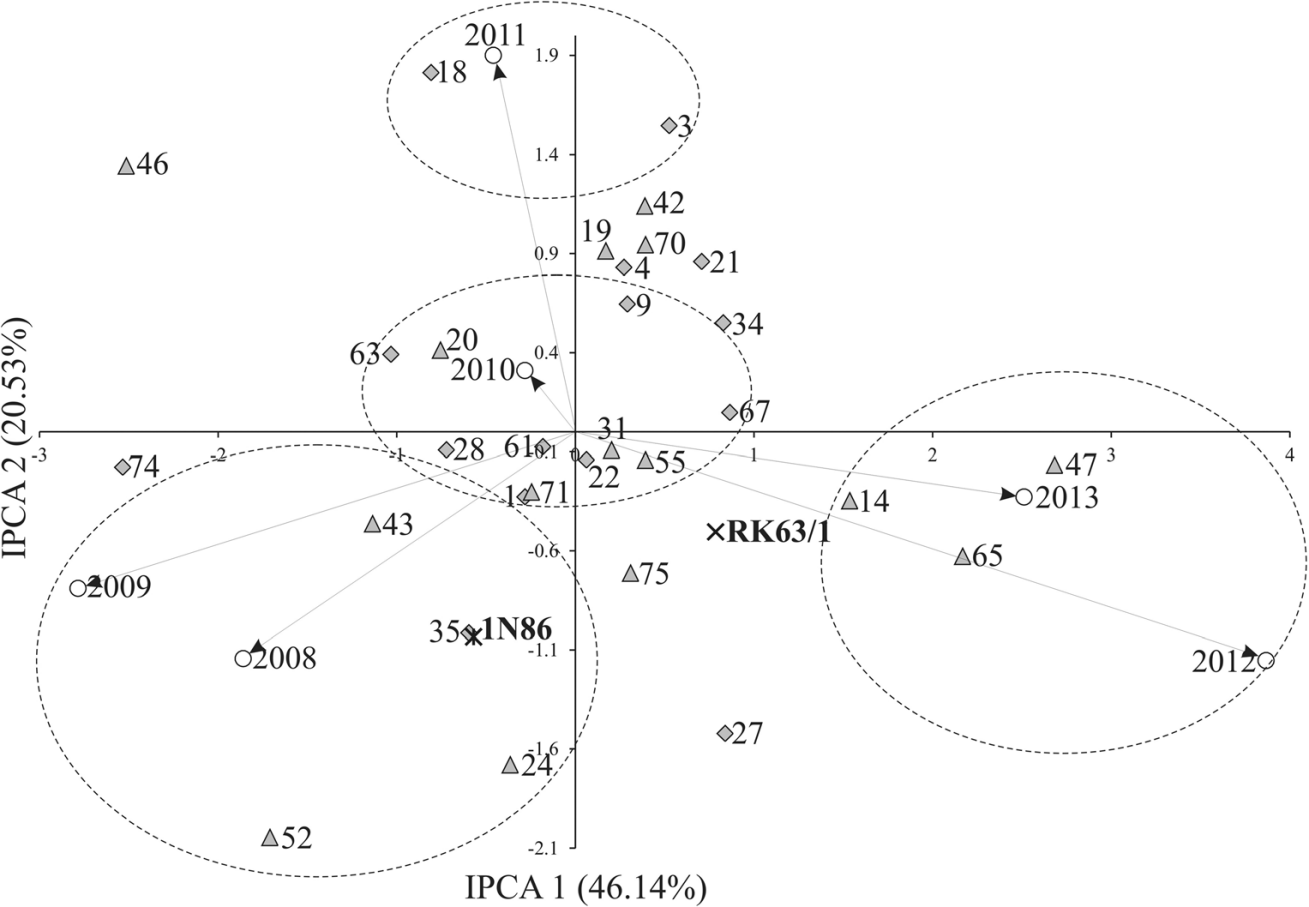
Biplot for genotype by environment interaction of spring barley (*Hordeum vulgare* L.) lines in six environments, showing the effects of primary and secondary components (IPCA1 and IPCA2, respectively)

In the analysis of variance, the sum of squares for genotype main effect accounted for 32.18% of the general sum, and this part had the maximum impact on 1000-kernel weight. The differences between environmental conditions clarified 16.86% of the total 1000-kernel weight variation, whereas the effects of GE interaction clarified 24.50% (Table 2). Values for the three major components were highly significant too. The three principal components of GE interaction possessed together 85.22% of the total effect it had on the variation of 1000-kernel weight. The first principal component (IPCA1) oscillated for 46.16% of the variation formed by interaction, IPCA2 oscillated for 20.53%, while IPCA3 oscillated for 18.55% (Fig. 1, Table 2).

**Table 2:** Analysis of variance of main effects and interactions for spring barley (*Hordeum vulgare* L.) lines 1000-kernel weight

Source of variation	d.f.	Sum of squares	Mean squares	*F*-statistic	Variability explained (%)
Genotypes	31	13,210	426.1	16.16***	32.18
Environments	5	6920	1384	15.84***	16.86
Interactions	155	10,057	64.9	2.46***	24.50
IPCA1	35	4640	132.6	5.03***	46.14
IPCA2	33	2065	62.6	2.37***	20.53
IPCA3	31	1866	60.2	2.28***	18.55
Residuals	56	1486	26.5	1.01	
Error	372	9810	26.4		

***P < 0.001. *IPCA*, principal component of interaction

The results of field trials demonstrated the impact of weather conditions, environment, and genotypes on the 1000-kernel weight of the spring barley genotypes. The 1000-kernel weight of the tested genotypes varied from 24.35 g (for R63N/42 in 2011) to 61.46 g (for R63N/18 in 2008), throughout the six seasons, with an average of 44.80 g (Table 3). The hulled line R63N/61 had the highest average 1000-kernel weight (54.59 g), and the hull-less line R63N/24 had the lowest (34.80 g). The average 1000-kernel weight per location also varied from 38.47 g in 2011, to 49.43 g in 2008.

**Table 3:** Average 1000-kernel weight (%), for genotypes and environments, principal component analysis values of tested spring barley (*Hordeum vulgare* L.) lines, AMMI stability value (ASV) and genotype selection index (GSI)

Line	Code	Type	2008	2009	2010	2011	2012	2013	Mean	IPCA1	IPCA2	ASV	GSI
R63N/1	1	Hulled	54.04	52.81	51.00	46.91	53.39	51.16	51.55	− 0.281	− 0.328	0.712	8
R63N/3	3	Hulled	59.91	43.82	52.20	37.56	56.44	48.36	49.72	0.527	1.546	1.947	27
R63N/4	4	Hulled	57.72	41.09	48.51	38.68	49.43	49.24	47.45	0.274	0.830	1.033	19
R63N/9	9	Hulled	52.20	38.26	47.51	34.63	42.49	51.03	44.35	0.292	0.645	0.921	26
R63N/18	18	Hulled	*61.46*	50.82	51.95	36.21	48.78	50.68	49.98	− 0.807	1.812	2.563	30
R63N/21	21	Hulled	56.13	50.74	53.78	42.04	58.61	56.47	52.96	0.708	0.859	1.807	19
R63N/22	22	Hulled	49.35	53.11	48.31	43.60	53.53	52.41	50.05	0.062	− 0.142	0.199	5
R63N/27	27	Hulled	41.54	38.48	45.35	42.96	46.21	47.60	43.69	0.839	− 1.524	2.425	45
R63N/28	28	Hulled	53.09	38.25	47.41	37.96	38.96	41.84	42.92	− 0.720	− 0.091	1.621	35
R63N/34	34	Hulled	50.88	41.50	49.86	37.22	51.18	50.67	46.88	0.828	0.550	1.941	32
R63N/35	35	Hulled	42.19	37.51	48.43	36.18	34.27	41.63	40.04	− 0.595	− 1.016	1.679	40
R63N/61	61	Hulled	55.29	56.65	55.70	47.35	55.04	57.52	*54.59*	− 0.180	− 0.076	0.412	3
R63N/63	63	Hulled	54.77	48.16	48.48	38.84	40.80	50.57	46.94	− 1.032	0.391	2.351	34
R63N/67	67	Hulled	53.26	33.62	51.18	38.03	44.57	51.62	45.38	0.866	0.097	1.949	38
R63N/74	74	Hulled	53.23	58.21	43.61	42.76	39.59	43.10	46.75	*− 2.534*	− 0.178	5.696	43
R63N/19	19	Hull-less	49.65	35.52	39.64	30.81	41.56	42.97	40.03	0.171	0.913	0.990	34
R63N/46	46	Hull-less	48.98	49.65	44.02	27.27	33.50	34.00	39.57	− 2.513	1.342	5.804	59
R63N/47	47	Hull-less	37.02	27.00	35.29	31.60	48.57	49.00	38.08	*2.684*	− 0.167	6.033	61
R63N/14	14	Hull-less	43.29	33.82	43.88	36.81	49.59	46.30	42.28	1.537	− 0.347	3.471	50
R63N/20	20	Hull-less	54.68	44.47	41.52	38.84	44.22	43.26	44.50	− 0.754	0.412	1.743	34
R63N/52	52	Hull-less	49.20	38.91	46.62	45.47	30.78	38.36	41.56	− 1.708	− 2.047	4.351	52
R63N/65	65	Hull-less	44.16	34.74	47.22	40.84	55.69	49.94	45.43	2.170	− 0.628	4.916	44
R63N/24	24	Hull-less	38.52	29.58	39.68	36.25	32.69	32.07	*34.80*	− 0.364	− 1.681	1.870	51
R63N/31	31	Hull-less	47.42	43.74	47.16	38.86	49.14	44.84	45.19	0.204	− 0.092	0.467	20
R63N/42	42	Hull-less	40.97	37.12	38.85	*24.35*	37.79	46.92	37.67	0.390	1.140	1.438	42
R63N/43	43	Hull-less	47.54	48.27	42.80	39.95	40.61	43.65	43.80	− 1.134	− 0.464	2.591	46
R63N/55	55	Hull-less	53.18	46.51	46.99	44.67	55.09	48.14	49.10	0.394	−0.142	0.897	13
R63N/70	70	Hull-less	53.80	48.18	47.81	38.81	55.20	49.96	48.96	0.394	0.944	1.294	19
R63N/71	71	Hull-less	50.55	44.51	43.71	41.25	44.35	48.73	45.52	− 0.244	− 0.303	0.627	18
R63N/75	75	Hull-less	37.32	34.51	35.56	32.59	35.79	43.25	36.50	0.310	− 0.714	0.997	40
RK63/1		Hulled	46.59	44.77	46.65	42.08	49.98	54.13	47.37	0.786	− 0.505	1.837	28
1 N86		Hull-less	43.87	40.02	38.48	39.54	39.47	38.56	39.99	− 0.569	− 1.035	1.645	41
Mean			*49.43*	42.64	45.91	*38.47*	45.54	46.81	*44.80*				
IPCA e[1]			− 1.859	*− 2.783*	− 0.281	− 0.458	*3.868*	2.513					
IPCA e[2]			− 1.145	− 0.791	0.310	1.900	− 1.154	− 0.329					

In italics are marked important values described in the “Results” section

**Fig. 2:**
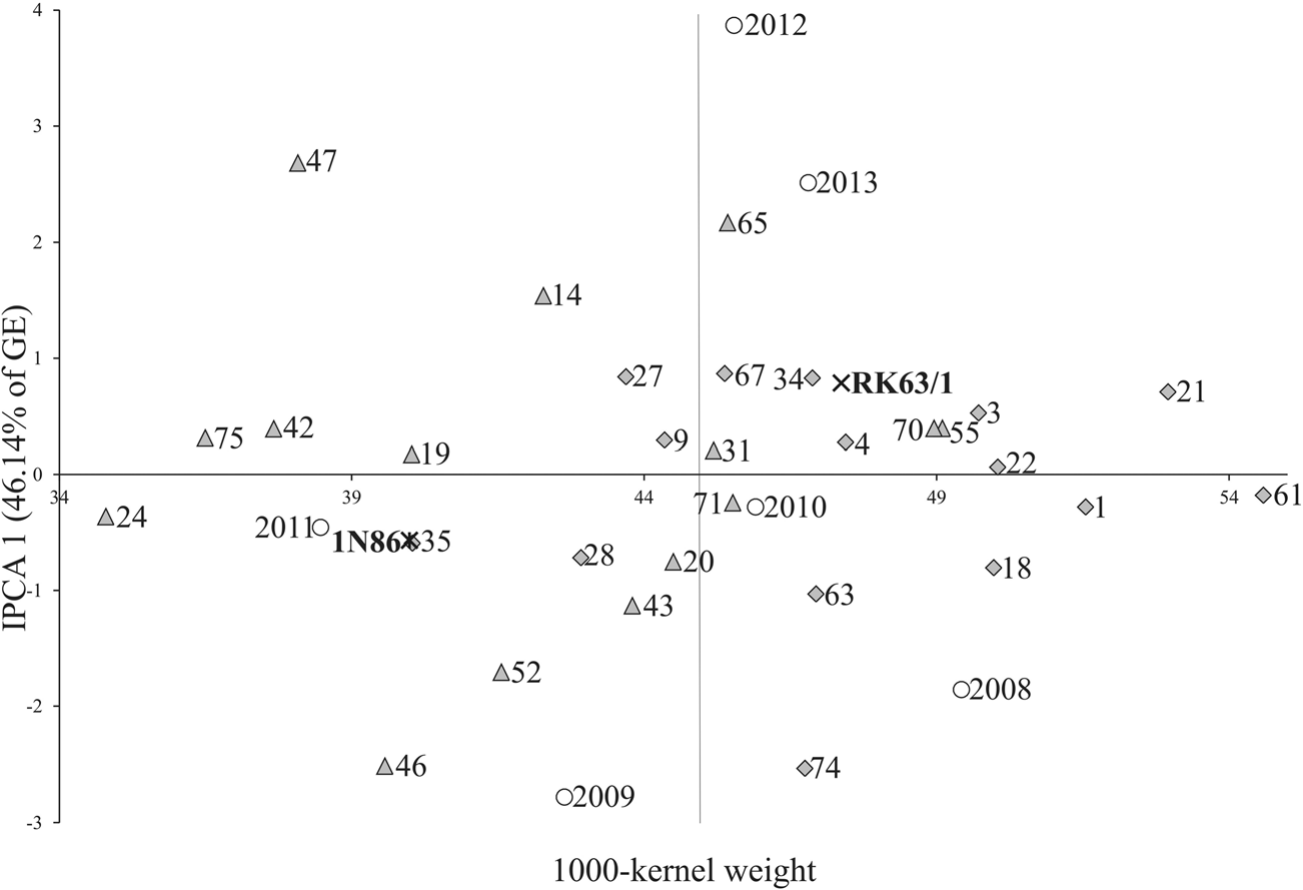
Biplot for the primary component of interaction (IPCA1) and average spring barley (*Hordeum vulgare* L.) 1000-kernel weight (g). Vertical line at the center of biplot is the general grand mean

The AMMI1 biplot (Fig. 1) shows the stability of genotypes and environments, as well as specific GE interactions. Among the tested genotypes, the hull-less line R63N/47 had the highest IPCA1 value of 2.684, while the smallest value of IPCA1 was − 2.534 for the hulled line R63N/74 (Fig. 1). Among the tested environments, the smallest IPCA1 value was observed in 2009 (− 2.783), while the highest value of IPCA1 was 3.868 in 2012 (Fig. 1). Genotype stability is considered as consistent reaction to changing environmental conditions, weather conditions, agronomic factors, and biotic and abiotic stresses. In this study, climatic conditions were the source of this variation component. The genotypes with specifically adaptation to certain environments were presented as four groups in Fig. 1. Meteorological conditions in the year 2012 and 2013 were similar according to temperature both in June and July but precipitation was much abundant in June 2013 with lower value in June 2012 (Table 1). The stability of tested genotypes can be evaluated according to biplot for 1000-kernel weight (Fig. 2). The lines R63N/14 and R63N/47 interacted positively with the years 2012 and 2013, but negatively with the years 2008 and 2009 (Figs. 1 and 2). Both lines positively react to high moisture and water recourses from high precipitation in June in the years 2012 and 2013 (Table 1) that is why a positive interaction with the years 2012 and 2013 was revealed. The water conditions were quite diverse in 2008 and 2009 in June (Table 1) and the negative interaction with the years 2008 and 2009 of the lines mentioned above were detected. It could be assumed that both lines react positively to high water availability in June, and high water potential impact positively 1000-kernel weight. The lines R63N/52 and R63N/74 interacted positively with the years 2008 and 2009, but negatively with the years 2012 and 2013. The lines R63N/20 and R63N/63 interacted positively with the year 2010. The lines possessed higher 1000-kernel weight when July was warmer than June and the water availability was similar in June and July since precipitation for both months were high and alike. The analysis showed that some genotypes have high adaptation; however, most of them have specific adaptability. AMMI stability values (ASV) revealed variations in 1000-kernel weight stability among the 25 genotypes (Table 3). According to Purchase et al. (2000), a stable variety is defined as one with ASV value close to zero. Consequently, the hulled lines R63N/22 and R63N/61 with ASV of 0.199 and 0.412, respectively, as well as hull-less line R63N/31 with ASV of 0.467 were the most stable, while the lines such as R63N/47, R63N/46, and R63N/74 were the least stable (Table 3). Genotypes on the highest point in certain sections of the graph have the best results in environments located in the same section (Fig. 2). The hull-less line R63N/20, with average 1000-kernel weight of 44.50 g close to the general mean of 44.80 g, is distinguished on the biplot. This line had the highest stability. A group of lines: R63N/61, R63N/21, R63N/1, R63N/22, R63N/18, and R63N/3, had the highest averages of 1000-kernel weight, but with different adaptations (Figs. 1 and 2): R63N/18 and R63N/1 showed specific adaptation to the conditions in 2011, and R63N/61, R63N/22, and R63N/1 showed the highest stability. These three hulled lines had the best genotype selection index 3, 5, and 8 respectively (Table 3).

Estimates of additive gene action effects for 1000-kernel weight based on DH lines were always larger (for all DH lines, for hulled lines, and for hull-less lines) the parameter estimated on the basis of parental lines (Table 4). Additive effect estimated on the basis of observation of parental lines was significantly larger than zero in 2009, 2010, 2012, and 2013 and for average of years (Table 4). Estimates of additive gene action effects based on the all DH lines were significantly larger than zero in each year of study. Only for average of years we observed additive effect of 1000-kernel weight non-significant larger than zero (Table 4). For hulled lines, additive gene action effects were significant in 2009 and 2012; however, for hull-less lines were significant in 2009, 2011, and 2012 (Table 4). Estimates of epistasis effects for 1000-kernel weight based on all DH lines were statistically significant in 2011 and 2013; for hulled lines in 2008 and 2012; and for hull-less lines in 2009, 2010, 2011, and 2013 (Table 4). All statistically significant epistasis effects were negative (Table 4).

**Table 4:** Estimates of additive and epistasis effects for 1000-kernel weight

Year	2008	2009	2010	2011	2012	2013	Mean
Parental forms							
*a*_Parents_^a^	1.36	2.38*	4.09*	1.27	5.26**	7.79**	3.69*
All lines							
*a*_*DH*_^b^	12.22*	15.61*	10.21*	11.50*	13.92*	12.73*	9.90
*aa*_*DH*_^c^	− 0.47	− 0.05	− 0.64	− 2.46**	− 0.90	− 2.05***	−0.18
Hulled lines							
*a*_*DH*_	9.96	12.30*	6.05	6.36	12.17*	7.95	7.28
*aa*_*DH*_	− 1.50**	0.38	0.10	0.93	− 1.11*	− 0.02	− 0.24
Hull-less lines							
*a*_*DH*_	8.83	11.33*	6.26	10.56*	12.46*	8.95	7.15
*aa*_*DH*_	− 0.57	− 1.44**	− 1.17*	− 1.65**	− 0.40	− 3.08***	− 0.25

*P < 0.05; **P < 0.01; ***P < 0.001

^a^The total additive effect estimated on the basis of parental observations

^b^The total additive effect estimated on the basis of doubled haploid lines observations

^c^The total additive-by-additive interaction effect estimated on the basis of doubled haploid lines observations

## Discussion

The 1000-kernel weight in spring barley (*Hordeum vulgare* L.) is a trait determined by multiple genes that cause change in the performance of genotypes depending on the cultivation environment. In this study, the three sources of variation were highly significant. Similar results for 1000-kernel weight in barley were obtained by Swanston et al. (1997) and Boudiar et al. (2016). Kumar et al. (2017) obtained not-significant GE interaction for 1000-kernel weight of 25 genotypes grown in India. Apart from the GE interaction, the most important information provided by multi-location experiments, the AMMI biplot, also give a chance to visualize the main genotype effect in different environments. Studies on GE interaction are of utmost importance and can be performed using several different methods, including linear-bilinear models, which offer an improved description of the effects of interaction among factors. The additive main effects and multiplicative interaction (AMMI) model is currently one of the most popular multiplicative models. The AMMI model was originally proposed by Gollob (1968) and Mandel (1969, 1971) in the context of fixed effects. In this paper, we used the traditional AMMI model for fixed effects. The study of many species frequently utilized the traditional AMMI model for fixed effects (Abakemal et al. 2016; Edwards 2016; Nowosad et al. 2016; Bocianowski et al. 2019a, b). Alternative to estimation of GE interaction is the REML/BLUP method, also known as the mixed model; it has great ability to explain GE interaction, to inform about specific positive or negative interactions with environments, and to decompose the interaction in terms of “pattern” or “noise” (Piepho et al. 2008; da Silva et al. 2015). The REML/BLUP method allows the consideration of different structures of variance and covariance for the genotypes × environments effects (Ferraudo and Perecin 2014).

The AMMI model was found as a constructive tool in indicating GE interaction patterns and improving the correctness of response estimates. It enables clustering of genotypes based on similarity of response characteristics and detecting possible trends throughout environments.

Researchers gain powerful tool in identifying definite cultivars with competitive yields across different environments while applying the suggested strategy which could extract more information from the GE interaction (Nowosad et al. 2018). The 1000-kernel weight expression in South Poland is the most influenced trait by genotype and environment main effects as well as GE interaction. The significance of environment main effect resulted mainly from differences in values of precipitation between June and July in certain years. Whereas the influence of the temperature on 1000-kernel weight was low, comparing to precipitation. The observed tendencies are in accordance with physiological processes connected with grain development and formation. Water deficit could disturb grain formation and as a consequence could reduce 1000-kernel weight as was observed by other authors (Kaczmarek et al. 1999; Warzecha et al. 2010, 2011).

Genotypes best matched for exact environmental conditions might be detected based on AMMI analyses which permits estimation of interaction effect of a genotype in each environment. Significant GE interaction of 1000-kernel weight was indicated with AMMI analysis application.

High genotypes stability is linked with the AMMI stability value. Determination of the main effect of the genotype, the environment, and the most meaningful GE interactions could be assessed based on the AMMI results displayed on GE biplot. The AMMI models are able to measure the weight of the environments, the genotypes, and their interactions using a value that measure genotype stability in all environments taking into account the 1000-kernel weight.

Assessment of genetic parameters has a considerable role in spring barley breeding (Pal et al. 2010; Bocianowski 2008, 2014; Bocianowski et al. 2016). The result reveals the significance of additive as well as epistasis gene effects of 1000-kernel weight in 2 years (2011 and 2013) of the research. Devaux (2003) proved that more than 95% of plants are haploids; therefore, they possess one chromosome of each pair; this means each gene is represented by on alternative version of gene, i.e., one allele. That information is critical and crucial for our statistical model. Since after chromosome doubling each of the gene possessed identical version of alleles, there could be assumed that the plants are completely homozygous. Taking into account that information, it was possible to assess significance of additive and epistasis effect excluding dominance effect, there were no heterozygous loci in the DH line population. The existence of epistasis has significant connotation for breeders in any improving program. The epistasis effect was significant and combined with non-significant additive effect for 1000-kernel weight for hull-less lines in 2010 and 2013. It means that this feature was probably controlled by genes with little individual effects but tough gene-by-gene interaction effects (Bocianowski 2012a, c, 2013a, b; Bocianowski and Nowosad 2015). Epistasis effects involved in the models proved that genetic of these traits is complex and polygenic (Lander and Schork 1994; Lefebvre and Palloix 1996; Hermisson et al. 2003; Crow 2010). Epistasis has been demonstrated for numerous features in a number of cultivars of following species: barley (Kularia and Sharma 2005; Bocianowski et al. 2016), corn (Melchinger et al. 1987; Li et al. 2016), sugar beet (Abbasi et al. 2015), rice (Matsubara et al. 2015), oilseed rape (Bocianowski et al. 2017), common wheat (Bnejdi and El Gazzah 2008; Jaiswal et al. 2016), and sorghum (Finkner et al. 1981).
